# Whole-genome sequencing provides novel insights into the evolutionary history and genetic adaptation of reindeer populations in northern Eurasia

**DOI:** 10.1038/s41598-023-50253-7

**Published:** 2023-12-27

**Authors:** Kisun Pokharel, Melak Weldenegodguad, Stephan Dudeck, Mervi Honkatukia, Heli Lindeberg, Nuccio Mazzullo, Antti Paasivaara, Jaana Peippo, Päivi Soppela, Florian Stammler, Juha Kantanen

**Affiliations:** 1 Natural Resources Institute Finland (Luke), Myllytie 1, 31600 Jokioinen, Finland; 2 Arctic Centre, University of Lapland, 96100 Rovaniemi, Finland; 3 NordGen—Nordic Genetic Resource Center, 1432 Ås, Norway; 4 Natural Resources Institute Finland (Luke), 71750 Maaninka, Finland; 5 Natural Resources Institute Finland (Luke), Paavo Havaksentie 3, 90570 Oulu, Finland

**Keywords:** Population genetics, Genetics, Genome

## Abstract

Domestic reindeer (*Rangifer tarandus*) play a vital role in the culture and livelihoods of indigenous people across northern Eurasia. These animals are well adapted to harsh environmental conditions, such as extreme cold, limited feed availability and long migration distances. Therefore, understanding the genomics of reindeer is crucial for improving their management, conservation and utilisation. In this study, we have generated a new genome assembly for the Fennoscandian domestic reindeer with high contiguity, making it the most complete reference genome for reindeer to date. The new genome assembly was utilised to explore genetic diversity, population structure and selective sweeps in Eurasian *Rangifer tarandus* populations which was based on the largest population genomic dataset for reindeer, encompassing 58 individuals from diverse populations. Phylogenetic analyses revealed distinct genetic clusters, with the Finnish wild forest reindeer (*Rangifer tarandus fennicus*) standing out as a unique subspecies. Divergence time estimates suggested a separation of ~ 52 thousand years ago (Kya) between the northern European *Rangifer tarandus fennicus* and *Rangifer tarandus tarandus*. Our study identified four main genetic clusters: Fennoscandian, the eastern/northern Russian and Alaskan group, the Finnish forest reindeer, and the Svalbard reindeer. Furthermore, two independent reindeer domestication processes were inferred, suggesting separate origins for the domestic Fennoscandian and eastern/northern Russian reindeer. Notably, shared genes under selection, including retroviral genes, point towards molecular domestication processes that aided adaptation of this species to diverse environments.

## Introduction

Reindeer (*Rangifer tarandus*), in the *Cervidae* family of ruminant mammals, inhabits tundra and boreal forest regions in northern Eurasia and North America. Within *R. tarandus*, various ecotypes, often termed subspecies, have been identified, such as tundra reindeer (or mountain reindeer) (*R. t. tarandus*), North American caribou (*R. t. caribou*), forest reindeer (*R. t. fennicus*) and arctic Svalbard reindeer (*R. t. platyrhynchus*), based on their biogeographic distributions, morphological characters and sedentary or migratory life-history strategies^1^. In Fennoscandia and the northernmost regions of Russia, they descend from wild tundra reindeer, while in more southern reindeer herding sites in Russia, including southern longitudes in Siberian regions, forest reindeer are managed as well^1^. The geographic distribution of wild and domestic populations and tundra and forest reindeer populations overlaps in some regions, and there are reindeer herding cultures, such as among the Evenki, where the coexistence of wild and domestic populations has promoted intentional crossbreeding of domestic and wild reindeer^2^. In other cultures such as among Nenets or Chukchi, or Finns, herders deliberately try to eliminate cross-breeding and keep the two different populations separate. Although hybridisation can be an important source of genetic variation for adaptation, for example, it may also have undesirable genetic effects on native phenotypes and fitness-related traits in wild populations^3^. For example, animals resulting from hybridisation between the wild forest reindeer and domestic tundra reindeer would not be accepted in the conservation policies of endangered wild forest reindeer in Finland.

Recently, the very first genetic *Rangifer* studies applying genome sequence data and bioinformatic methods were published^4–7^ and currently there are de novo genome assemblies available for the Eurasian *R. t. tarandus*^4,7^ and North American *R. t. caribou*^6,8^. The recent de novo reference genomes of *R. t. tarandus* were assembled using Illumina technology sequence data^4,7^. However, the traditional Illumina mate-pair libraries may not span all genomic repetitive elements, resulting in less optimal, fragmented assemblies resulting into a high number of scaffolds^9,10^. To create fewer and longer scaffolds even at the subchromosomal level and to obtain a more contiguous assembly, chromosome conformation capture (3C) techniques, such as Hi-C and Chicago libraries, are available^11^. The improved de novo genome assembly is a critical tool in various genomic studies examining genome architecture, genomic diversity, associations between genomic and phenotypic data as well as evolutionary and population genomics^8,10–12^. The 3C approaches can also be used to detect fundamental units of three-dimensional genomic architecture, such as topologically associated domains (TADs), which play an important role in the gene expression regulations^13^.

The very first studies focusing on population genomics of the *Rangifer* were conducted applying the new de novo assemblies and whole genome resequencing data to examine within- and between-population diversity^6–8^, the research issues of which have previously been investigated using mitochondrial DNA and autosomal microsatellites as genetic markers^2,14,15^. Autosomal microsatellite, mitochondrial DNA datasets and whole genome resequencing have indicated relatively high genetic variation within domestic reindeer populations, which may be indicative of the early phase of reindeer domestication and breeding history and which, in a few cases, can also suggest events of introgression from wild populations^2,7,15^. Genome datasets have provided novel and versatile insights into the evolution, demographic history and spread of reindeer populations from refugia after the Last Glacial Maximum and effects of natural selection on several crucial genes, which have promoted the adaptation of reindeer to challenging northern environments^5,7^. The *PRDM9* gene, for example, has been involved in recombination and speciation, *PRDM1* and *OPN4B* in retinal development and *GRIA1* in circadian rhythm^7^.

In the present study, we promote the new research field of *Rangifer* genomics by describing our efforts to improve the current Fennoscandian reindeer reference genome^7^ and by publishing the largest population genomic data on Eurasian *R. tarandus* so far. We used Chicago and Hi-C sequencing libraries and HiRise pipeline for the reindeer genome assembly to produce long scaffolds. We applied the improved assembly for investigations of genetic diversity, population structure and selective sweeps in northern Eurasian domestic and wild reindeer populations. We updated our resequencing data, currently including genome sequences of 58 individuals and new sequence data of Fennoscandian (Finland), Nenets (Arkhangelsk in western Russia) and Eveny (Sakha Republic, the Russian Federation) domestic reindeer and wild forest reindeer (*R. t. fennicus*) in Finland. We conducted the most comprehensive population genomic studies for reindeer populations from which several individuals were resequenced.

## Results

### A new Fennoscandian reindeer reference genome

**Figure 1:**
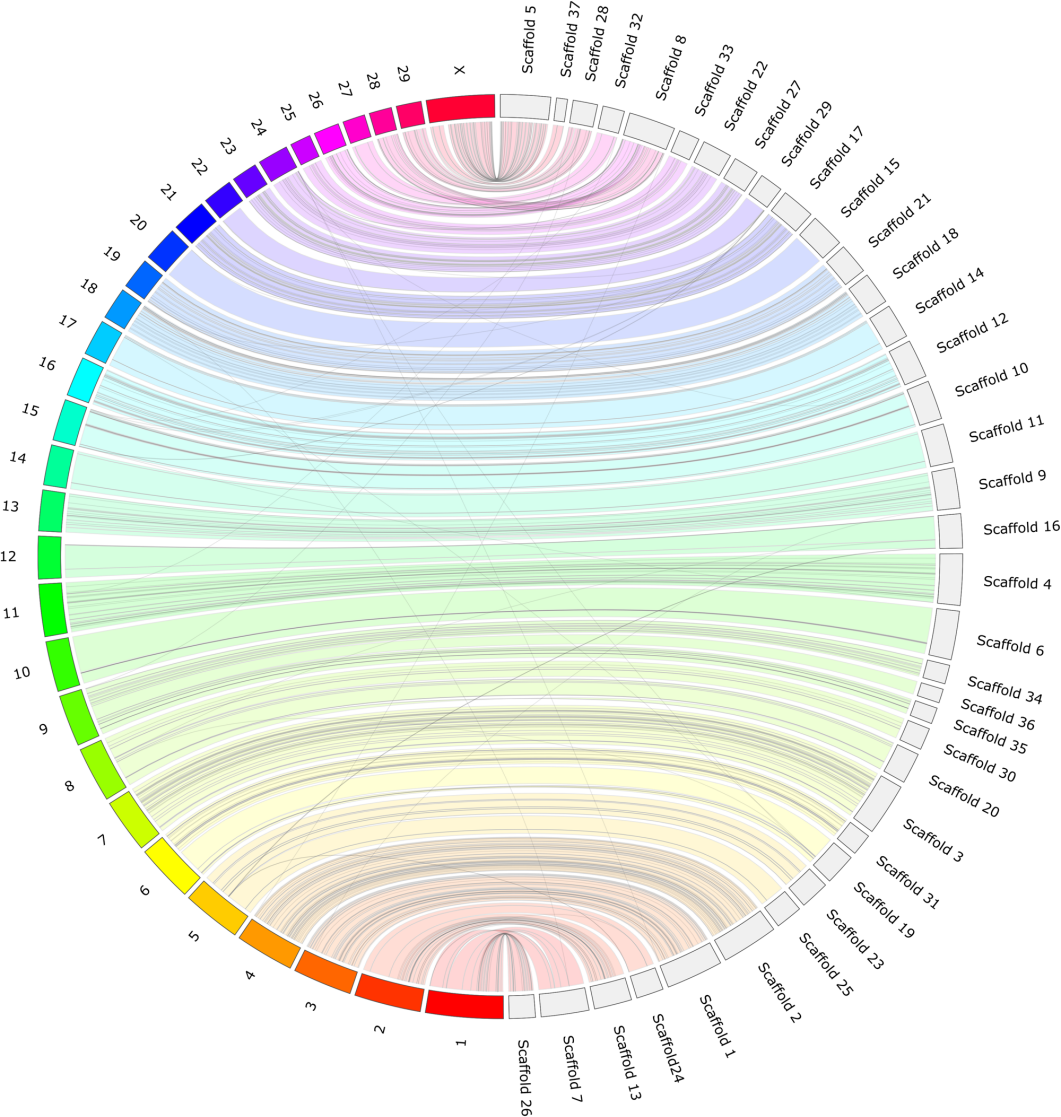
Jupiter consistency plot showing genome alignment between the reindeer assembly and cattle reference genome. The top 37 scaffolds (all above 10 Mb) represented 95% of the reindeer assembly and covered the entire 30 (29 autosomes and Chr X) chromosomes of the cattle reference genome. Coloured bands represent synteny between two genomes, and the crossing lines indicate possible genomic rearrangements or break points in the scaffolds.

We generated a new reference genome assembly of the domestic Fennoscandian reindeer derived from the same male reindeer individual, the genome of which was sequenced to produce the first reference assembly, including the mitochondrial genome^7^. To obtain the present new reference assembly, we used these recent data based on the Illumina shotgun-sequencing technology and the present new sequence data obtained by sequencing Chicago and Hi-C proximity ligation libraries. The number and length of read pairs produced for Chicago library 1 was 223 million and 2 × 150 bp, respectively, and for library 2 180 million and 2 × 150 bp, respectively. Together, these Chicago library reads provided 93.85 × physical coverage of the genome (1–100 kb pairs). For the Hi-C proximity ligation libraries, the corresponding values were as follows: 203 million and 2 × 150 bp for library 1; and 212 million and 2 × 150 bp for library 2. Together, these Dovetail Hi-C library reads provided 44,243.23 × physical coverage of the genome (10–10,000 kb pairs). The final assembly was comprised of 2663.35 Mb with a total of 127,880 scaffolds, of which 11,813 were greater than 1 kb. The longest scaffold in our assembly is 116,325,531 bp, and the N50 and N90 scaffold lengths are 38.508 Mb (L50 = 15 scaffolds) and 69.739 Mb (L90 = 34 scaffolds), respectively (Table 1, Supplementary Fig. 1). So far, five studies have reported genome assemblies for *R. tarandus* originating from different geographic regions (Table 1). In comparison with the metrics of existing assemblies, our new reference genome has fewer and longer scaffolds, thus indicating a good alternative reference for reindeer genomic studies. Moreover, synteny comparison showed that the top 37 scaffolds (all above 10 Mb) represent 95% of the reindeer assembly and cover the entire 30 (29 autosomes and Chr X) chromosomes of the cattle reference genome (Fig. 1). Cattle chromosomes 1, 2, 6, 8, 9 and X split into two scaffolds, each of reindeer, whereas cattle Chr 27 and 28 were represented by one reindeer scaffold. There were a few nonsyntenic regions, marked by intersecting lines/bands.

**Table 1:** Comparative summary statistics of existing reindeer genome assemblies with our new assembly.

	Li et al.^4^	Taylor et al.^6^	Weldenegodguad et al.^7^	Prunier et al.^8^	Poisson et al.^16^	This study
Total length (Gb)	2.64	2.21	2.66	2.59	2.60	2.66
No. of scaffolds	58,765	4,699	131,360	13,994	12,263	127,880
Contig N50 (Kb)	89.7	32.82	48.8	50.16	168.8	146,78
Scaffold L50/N50 (Mb)	NA 0.986	52 scaffolds; 11.765	157 scaffolds; 5.024	131 scaffolds; 29.299	18 scaffolds; 54.4	15 scaffolds; 69.739
Scaffold L90/N90 (Mb)	NA NA	289 scaffolds; 0.897	624 scaffolds; 0.839	NA NA	94 scaffolds NA	34 scaffolds; 38.508
No. of genes	21,555	33,177	27,332	17,394	NA	32,721

*NA* not available.

The genome contained 35.52% of repeats, of which Class I transposable element (TE) repeats comprised of 30% and Class II TE repeats were 1.71% (TEs reviewed in, e.g. Lerat^17^). Altogether 32,721 genes were present in our assembly, with the total coding region spanning 32,201,636 bp (% of genome) with an average length of 984 base pairs. There were a total of 1,694 single-exon genes. Benchmarking Universal Single Copy Orthologue (BUSCO) analysis^18^ revealed 202 complete single-copy BUSCOs, and 18.8% were missing. We manually curated 55 genes (Supplementary Data 1), of which seven could not be found.

Altogether, 263 TADs were detected at 10 kbp resolution, and with 50 kbp resolution, we observed 1452 TADs (Supplementary Table 1). Those 263 TADs detected with the resolution of 10 kbp represented 6.44% of the genome with an average TAD size of 645,741 bp. Moreover, the number of isochores and CCCTC-binding factor (CTCF) sites was 23,575 and 11,077, respectively. Research on TADs and their functional significance in genome architecture is still in its early stages, even in the context of human studies. Nevertheless, given that TADs have the potential to contribute to the heritability of complex traits, including metabolic and immunological traits^19^, they could serve as important markers for future research on livestock animals to understand adaptation mechanisms and their implications for breeding practices.

### Population genomic analysis

#### Genomic variants

For the present study, 35 individuals of the Finnish, Nenets and Eveny domestic reindeer and the wild Finnish Forest reindeer were resequenced (Supplementary Data 2). After the new raw resequenced data were processed, we generated a total of 1.57 Tb clean paired-end new data. In our previous *Rangifer* whole genome data comprising 23 animals^7^ and pooled here to the present new data, we generated 680 Gb clean paired-end whole genome sequencing data. Alignment of the reads to the present assembled reference genome rta_v2.0 was successful, with 98.7% of the reads mapped to the reference genome on average per individual, indicating that the qualities of the whole genome sequencing data were found to be sufficiently good for downstream analyses. The average sequencing coverage of the 58 genomes was 11.4 x, varying from 8.1 to 15.8 × coverage (Supplementary Data 2). Average depth was lower, with slight variation in the older sample batch (NMBU-* samples) and the newer sample batch having higher depth of coverage on average but greater variation in depth between the samples.

A total of 41.09 million high-quality single nucleotide polymorphisms (SNPs) were detected across all 58 individuals (the pooled data). The average number of SNPs detected per individual was 8.41 M (Supplementary Fig. 3B). A total of 5.8 M indels were also detected across all samples, with average indel count per individual being 1.2 M (Supplementary Fig. 3C). Of the 12 different populations, the Eveny domestic reindeer from the Sakha (Yakutia) Republic (nine animals) showed the highest number of SNPs, with 20.8 M SNPs found in total among the population and 9.38 SNPs detected on average per individual. At the other end of the spectrum, the Svalbard wild arctic reindeer population (only three individuals) contained a total of 8.59 M SNPs, with the average number of SNPs identified per individual being 7.17 M. Noteworthy is the split of the Nenets domestic reindeer from Arkhangelsk into two groups, with SNP counts of 6.31–6.98 M in six individuals and SNP counts of 8.43–9.34 M in four individuals. Indel counts followed the same general trend, but on visual inspection of the plots, this trend appeared not to have a clear correlation with the sequencing depth. Also, the transition to transversion (Ts/Tv) ratios were uniform and within the expected range across all the samples (Supplementary Fig. 4A; Supplementary Data 2) and populations (Supplementary Data 3), indicating consistent quality of the call set. The proportions of homozygous and heterozygous SNPs in the Svalbard wild arctic reindeer population clearly deviated from the other populations, showing a skew towards homozygous SNPs; these results are in line with the Svalbard population being an isolated population with presumably high inbreeding (Supplementary Fig. 4B).

### Genetic relationships between 59 animals

**Figure 2:**
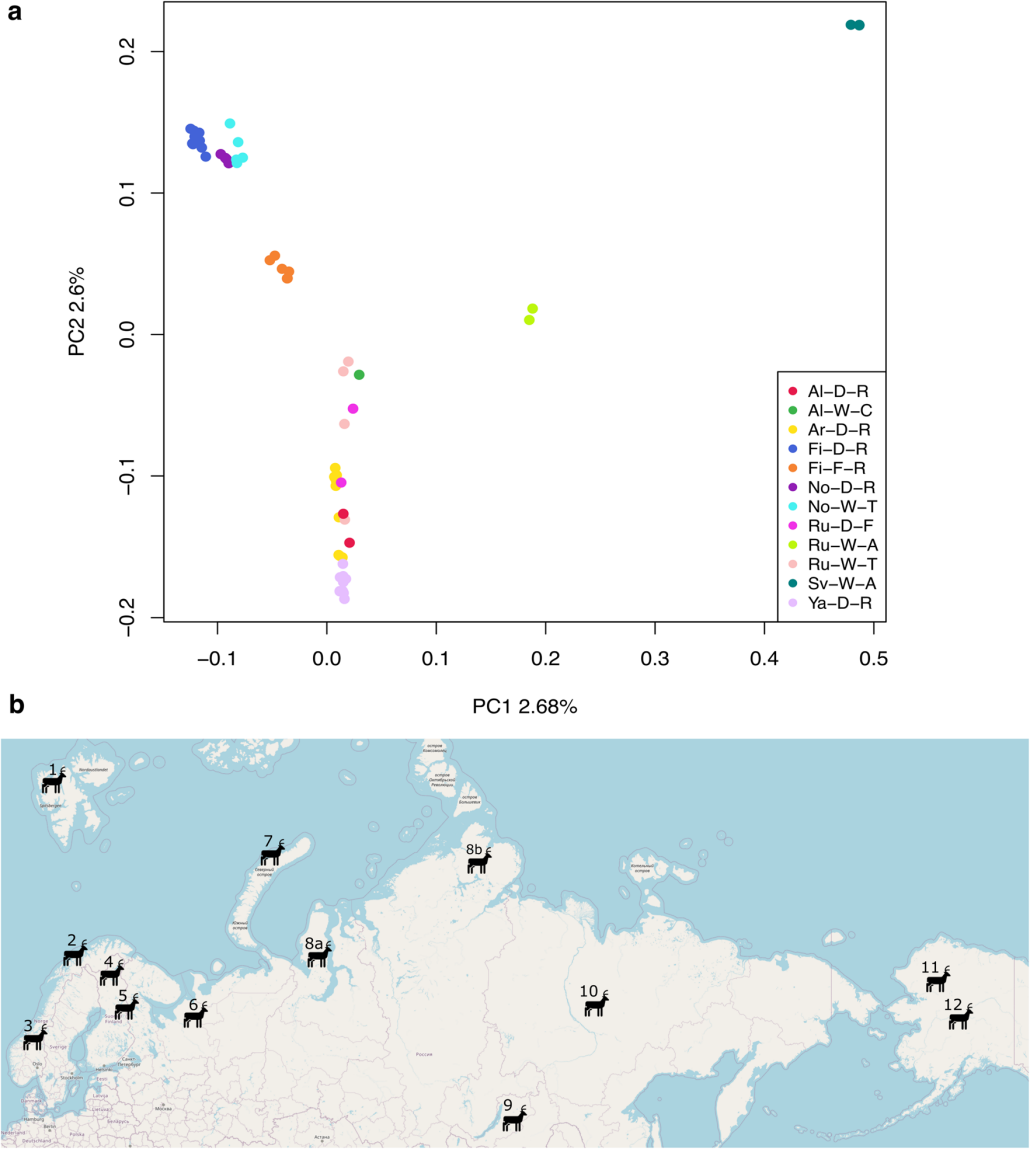
Sample distribution. (**a**) Principal component analysis (PCA) plot based on filtered and LD-pruned SNPs of all 59 samples. (**b**) Geographic sites of reindeer populations included in this study. The 12 main populations are as follows: (1) Svalbard wild arctic reindeer (Sv-W-A – Svalbard, Norway): (2) Norwegian domestic tundra reindeer (No-D-R – Finmark and Filefjell, Norway); (3) Norwegian wild tundra reindeer (No-W-T – Hardangervidda, Norway); (4) Finnish domestic reindeer (Fi-D-R – Ivalo and Inari, Finland); (5) Finnish wild forest reindeer (Fi-F-R – Kuhmo, Finland); (6) Nenets domestic reindeer (Ar-D-R – Arkhangelsk, Russia); (7) Russian wild tundra-mountain reindeer (Ru-W-A – Novaja Zemlya, Russia); (8)—Russian wild tundra reindeer (Ru-W-T – Yamal and Taymyr, Russia); (9) Russian domestic forest reindeer (Ru-D-F – ZaiBaikal, Russia); (10) Eveny domestic reindeer (Ya-D-R – Yakutia, Russia); (11) Alaskan wild caribou (Al-W–C – Alaska, USA); (12) Alaskan domestic reindeer (Al-D-R – Alaska, USA).

Genetic relationships between all the 59 animals, including the reference animal, were studied using principal component analysis (PCA). The PCA plot of the SNP data (Fig. 2) displayed that the three Fennoscandian tundra reindeer populations (i.e. the Finnish domestic reindeer, Norwegian domestic reindeer and Norwegian wild tundra reindeer) were in the same cluster and that the Russian and Alaskan animals formed a more heterogenous cluster separated by the second principal component from the Fennoscandian tundra populations and distinct clustering of the Svalbard wild arctic reindeer and Russian wild tundra reindeer from Novaja Zemlya based on the first principal component. Moreover, the Finnish wild forest reindeer was found in a distinct cluster separated by the second principal component from the two main clusters.

**Figure 3:**
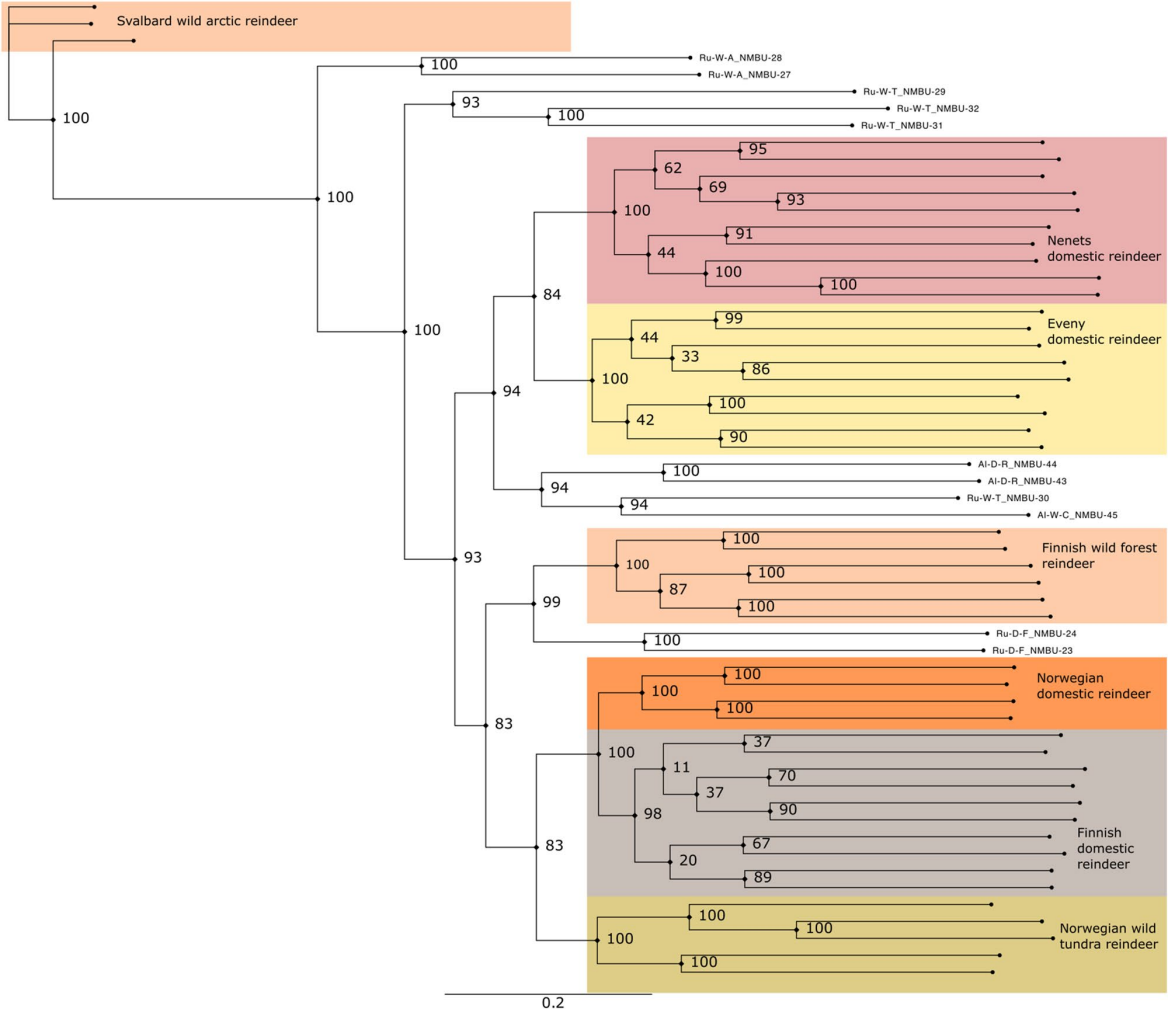
Genetic relationships between 59 animals. Neighbour-joining (NJ) tree constructed to show genetic relationships between 59 animals, calculated from the single nucleotide polymorphism (SNP) data. Bootstrap confidence values obtained from 100 bootstrap replicates are shown at each branch. Seven main populations are highlighted by colour.

The genetic relationships of the individual reindeer were further studied by constructing a neighbour-joining (NJ) tree based on SNP data (Fig. 3). As with the PCA analysis, the populations formed distinct clusters. Two main phylogenetic clusters – the *Rangifer* of Fennoscandia and the *Rangifer* of the eastern/northern Russian Federation and Alaska – were identified with high bootstrap confidence values. Within these major clusters, animals tended to group according to their known ancestries and geographic origins, such as the Nenets domestic reindeer in Arkhangelsk, the Eveny domestic reindeer in Sakha, the Finnish wild forest reindeer, the Norwegian domestic reindeer, the Finnish domestic reindeer and the Norwegian wild tundra reindeer. Interestingly, in the phylogenetic tree, the domestic forest reindeer from the Zabaikal region in southern Siberia (see Andersson et al.^2^) grouped to the same branch with the Finnish wild forest reindeer, with a high bootstrap value. Moreover, the Svalbard wild arctic reindeer appeared clearly more distant to all the other populations.

**Figure 4:**
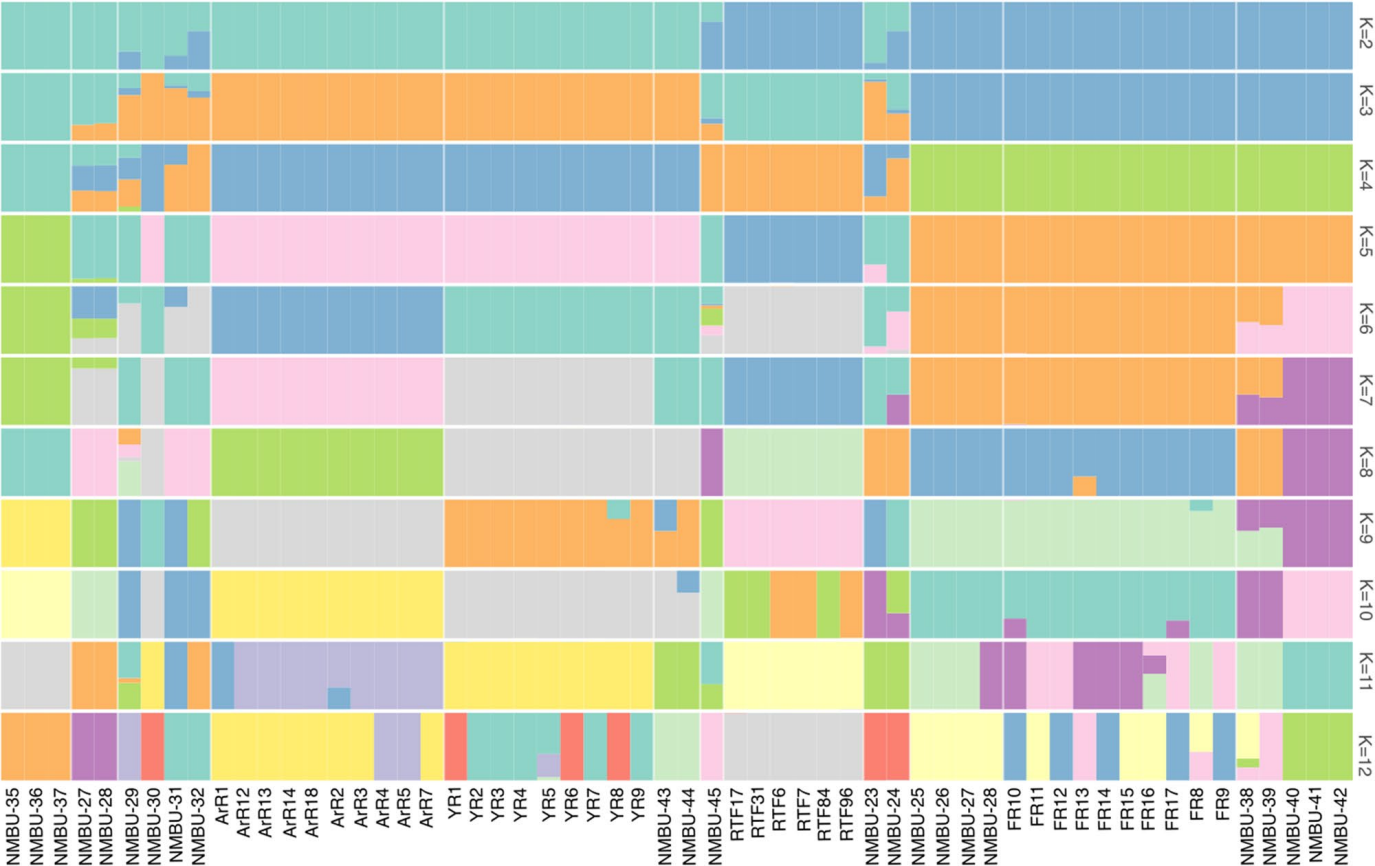
Population structure analysis of the twelve populations using ADMIXTURE. The bars represent individuals in a population and are segmented into colours based on the cluster assignment. The estimated proportion of the individual’s genome that belongs to a given cluster is indicated by the length of the coloured segment. The analysis was repeated with different assumed numbers of clusters (K) that are indicated on the y-axis. Population codes and domestication status of the individuals are indicated on the x-axis. Individuals have been sorted within the population based on the cluster assignment values.

To infer population structure and admixture, ADMIXTURE software (Version 1.3) was used for population structure analysis, including 58 animals from 12 populations, using K values ranging from 2 to 12 (Fig. 4). Cross-validation errors of the cluster numbers were lowest for K values 2, 3 and 4, increasing after that as K increased (Supplementary Fig. 7). This and the PCA plot suggest that four would be the optimal number of ancestral populations. From the structure plots, one can, for example, see that Svalbard wild arctic reindeer are the only animals that are consistently assigned to their own unique cluster across all K (excluding K = 2 and 3) values. As indicated in the plot, K = 2 splits reindeer populations of Fennoscandian origin from the other populations. At K = 3, the Finnish wild forest reindeer (Fi-F-R) showed distinctiveness from the Fennoscandian wild and domestic tundra reindeer. At K = 4, the three Fennoscandian populations Fi-D-R, No-D-R and No-W-T were assigned to a separate cluster from the Eveny and Nenets domestic reindeer, while the Svalbard wild arctic reindeer and Finnish wild forest reindeer formed the two other clusters. Here, the two domestic forest reindeer from the Zabaikal region showed admixture of the Finnish wild forest reindeer and the Russian populations. In general, the clustering is well in line with the results of the PCA and NJ tree results (Figs. 2 and 3, respectively).

### Genetic analysis of seven *Rangifer* populations

Based on the PCA, topology of the phylogenetic tree, geographic origins and the number of sequenced animals, seven ‘main’ populations were selected for further within- and between-population genetic studies (see Table 2).

**Table 2:** Population diversity statistics.

Population	n	π (10^–3^)	θ (10^–3^)	No. of SNPs	No. of indels	Ts/Tv ratio	Het. variants	Hom. variants
Finnish domestic reindeer	10	2.35	2.32	20,503,512	3,310,423	1.9	5,480,343	3,046,255
Norwegian domestic reindeer	4	2.02	1.96	13,939,267	2,061,972	1.91	4,359,560	3,135,789
Norwegian wild tundra reindeer	5	2.15	2.1	16,054,462	2,340,867	1.92	4,144,039	3,903,346
Finnish wild forest reindeer	6	2.27	2.19	17,836,068	2,911,350	1.89	5,619,178	3,617,497
Nenets domestic reindeer	10	2.26	2.02	19,184,388	3,100,437	1.89	4,198,342	2,587,754
Eveny domestic reindeer	9	2.33	2.29	20,798,132	3,361,007	1.9	5,572,380	3,671,050
Svalbard wild arctic reindeer	3	0.742	0.694	8,590,785	1,345,633	1.85	1,757,971	5,467,437

Here, n represents the number of samples in each of the seven populations. Other statistics presented in the table are nucleotide diversity π, Watterson’s θ, the number of single nucleotide polymorphisms (SNPs) and indels, transition to transversion (Ts/Tv) ratios and heterozygous and homozygous SNPs.

### Population diversity statistics

Genetic diversity parameters were calculated for the reindeer populations based on the SNP data that was filtered with linkage disequilibrium (LD) threshold 0.1 and MAF threshold 0.05 (totalling 14.9 million SNPs). The two main measures of overall genome-wide genetic diversity, pairwise nucleotide diversity (π) and expected level of diversity (Watterson’s θ), within the populations are shown in Table 2. The lowest π and θ values were, as expected, in the Svalbard population (0.742 × 10^–3^ and 0.694 × 10^–3^, respectively), while the highest values were found within the Finnish domestic reindeer (2.35 × 10^–3^ and 2.32 × 10^–3^, respectively). Moreover, numbers of private (i.e. population-specific) variants were also compared between the populations. The Svalbard reindeer and Norwegian domestic reindeer had the smallest proportions of population-specific variants (only four animals were sequenced in the Norwegian reindeer), whereas the highest proportion of population-specific variants was found in the Finnish domestic reindeer (Table 2).

A population-level phylogenetic analysis was also conducted for the seven populations. For that, pairwise F_ST_ values were calculated and were used as the distance metric for building a NJ tree. The lowest pairwise F_ST_ was found between the Finnish and Norwegian domestic reindeer, namely 0.009113, indicating a low differentiation between the populations (Supplementary Table 3). The highest F_ST_ values were found when the Svalbard wild arctic reindeer was compared to other populations, the values ranging from 0.388949 (comparison to Eveny domestic reindeer) to 0.424133 (comparison to Norwegian domestic reindeer). As with the individual-level NJ tree (Supplementary Fig. 6), the Svalbard population is clearly distinct from the other populations. There is also a clear separation of the Eveny and Nenets domestic reindeer populations (from Yakutia and Arkhangelsk, respectively) from the four Fennoscandian populations. F_ST_ values between one population and all the others were also determined using a sliding window approach, dividing all analysed SNP sites into 10 K SNP windows. Average F_ST_ values within each window were then plotted (Supplementary Fig. 7).

### Signatures of selection

In order to perform genome-wide scans for selective sweeps, we focused on reindeer populations identified in the PCA (Fig. 2) and phylogenetic (Fig. 3) analysis and carried out selective sweep analysis using RAiSD software^20^ in five subpopulations, namely Finnish wild forest reindeer, Norwegian wild tundra reindeer, Fennoscandian domestic reindeer (the Finnish and Norwegian populations pooled), Nenets domestic reindeer and Eveny domestic reindeer (Supplementary Data 4). We found many genomic regions exhibiting selective sweeps in each population – a total of 1538 in the Finnish wild forest reindeer, 1352 in the Norwegian wild tundra reindeer, 1928 in the Fennoscandian domestic reindeer, 1197 in the Nenets domestic reindeer and 1832 in the Eveny domestic reindeer – distributed in the top 40 scaffolds. These regions show significantly higher values of μ-statistics due to a result of positive selection, as μ-statistics are a measure of positive selection. The identified selective sweep genomic regions were mapped to several genes: 247 (Finnish wild forest reindeer), 290 (Norwegian wild tundra reindeer), 258 (Fennoscandian domestic reindeer), 176 (Nenets domestic reindeer) and 271 (Eveny domestic reindeer) (Supplementary Data 4).

In our investigation, we found several genes related to cold adaptation, such as non-shivering thermogenesis, smooth muscle contraction, blood pressure, response to temperature, basal metabolic rate and energy metabolism^21^, which were under positive selection in the Finnish wild forest reindeer (*CKMT2*, *EDN3* and *HSPB6*), the Fennoscandian domestic reindeer (*GCLM*), the Nenets domestic reindeer (*DNAJC1*, *DNAJC11* and *KCNB1*) and the Eveny domestic reindeer (*AHR*, *DNAJC11*, *GNAS* and *HR*). Moreover, the identified selective sweep genes in the populations include genes associated with immune response, for instance in the Finnish wild forest reindeer (*LY9, FGB, TRAV12-3, TRAV14DV4, TRAV22* and *TRAV41*), the Norwegian wild tundra reindeer (*ALOX15*, *CD3D*, *CD3G*, *ATF7*, *IFITM1*, *IFITM2*, *IFITM3*, *KLRC1*, *TGFB1*, *TRAV26-2*, *TRAV27*, *TRAV8-3*, *TRAV9-1* and *TRDV1*), the Fennoscandian domestic reindeer (*IRF8, MAP3K8, NFKB2, TRAV10, TRAV13-1, TRAV14DV4, TRAV26-2, TRAV8-3, TRDV1* and *TRIM5*), the Nenets domestic reindeer (*SEMA4D, TRAV22, TRAV24, TRAV26-1, TRAV26-2, TRAV27, TRAV8-3* and *TRDV1*) and the Eveny domestic reindeer (*AHR, ATRN, IFITM1, IFITM2, IFITM3, NLRP6, PLXNC1, TRAV12-3, TRAV14DV4, TRAV22, TRAV24, TRAV41* and *TRIM5*).

We also identified a number of genes under selection in the *Rangifer* population associated with ATP, lipid and energy metabolism, such as *APOO*, *CD5L, CD1E, ATP8B1, CKMT2, GABBR1* and *LIPK* in the Finnish wild forest reindeer, *CD5L, NUDT5, PERM1, P2RX7, SCP2* and *VPS9D1* in the Norwegian wild tundra reindeer, *CD5L, CYP1B1, DNAH2, GNPAT, PERM1* and *SCP2* in the Fennoscandian domestic reindeer, *CD5L, FGGY, SCP2* and *SMCHD1* in the Nenets domestic reindeer and *APOM, ATP6V0A1, CD5L, PERM1* and *SCP2* in the Eveny domestic reindeer.

In addition, we found several genes under positive selection associated with calcium binding, calcium metabolism and calcium homeostasis, and circadian rhythm. Previous studies have shown that genes associated with calcium metabolism and circadian rhythm are found to be associated with reindeer specific characteristics^5^. Examples of these genes associated with calcium binding, calcium metabolism and calcium homeostasis are *GABBR1*, *KCNIP1* and *NINL* in the Finnish wild forest reindeer, *CACNA1E*, *CACNA2D2, GABBR1, PCDHA2, PCDHA9* and *SLC35G1* in the Norwegian wild tundra reindeer, *OPRL1, PCDHA2, PCDHA5, PCDHA6, PCDHA7, PCDHA9, PCDHB15* and *VAPB* in the Fennoscandian domestic reindeer, *ADGRL2, PCDHA11, PCDHA5, PCDHA6, PCDHA7, PCDHB15* and *PCDHB18* in the Nenets domestic reindeer and *CDH15* in the Eveny domestic reindeer. Moreover, among the identified ‘selective sweep genes,’ there were genes know to be associated with circadian rhythm: *NFKB2* in the Fennoscandian reindeer and *AHR*, *DRD4* and *HCRTR1* in the Eveny reindeer^22–28^.

**Figure 5:**
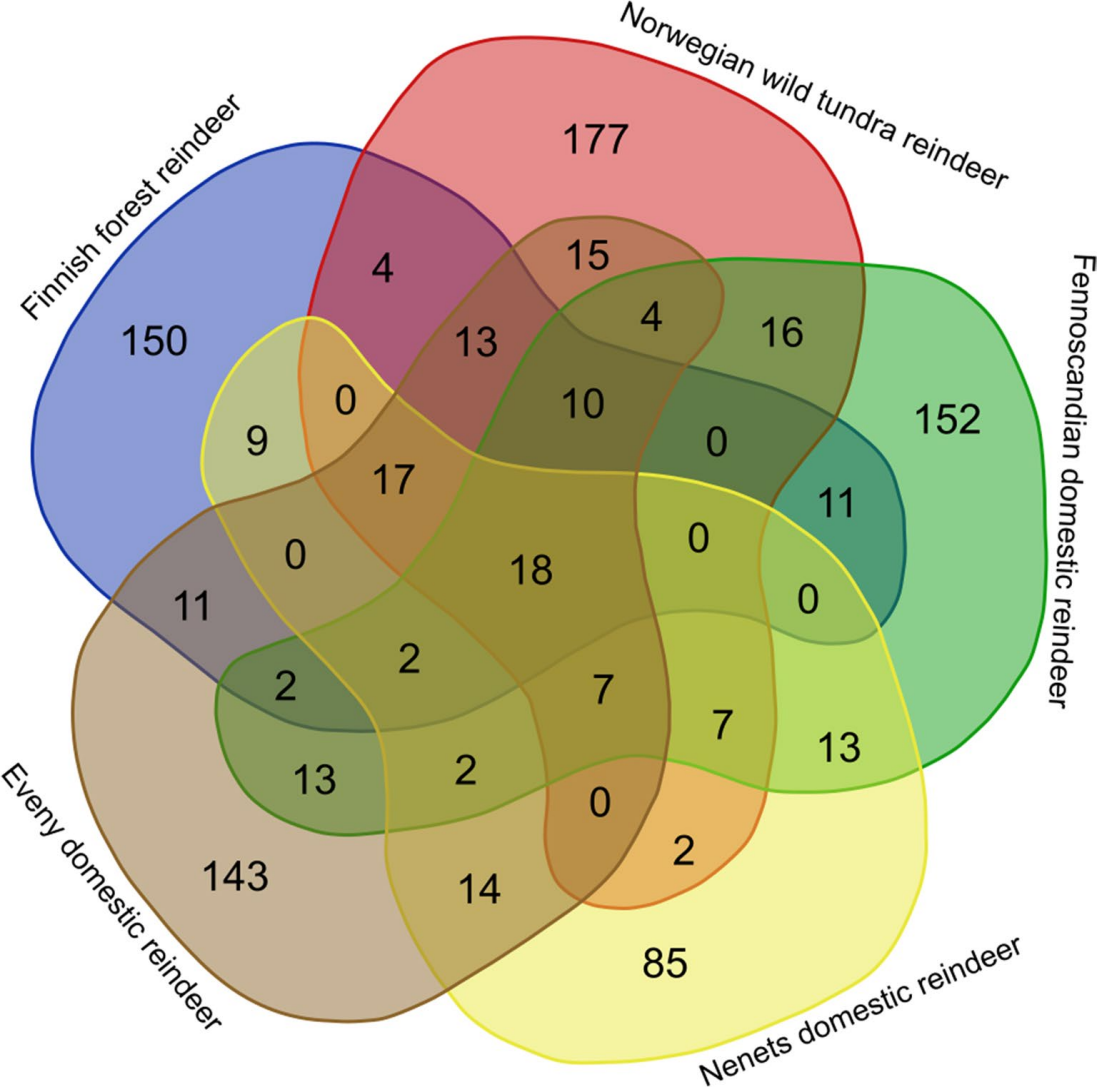
Distribution of genes under selection in five major groups: the Finnish forest reindeer, the Norwegian wild tundra reindeer, the Fennoscandian domestic reindeer, the Nenets domestic reindeer and the Eveny domestic reindeer.

We looked for common genes under selection across all reindeer populations, as well as those unique to each of the five populations (Supplementary Data 5). As shown in the Venn diagram (Fig. 5), 18 genes were found to be under selection in all populations. These common genes included *Gag*, *Pol*, *RAB9A*, *MAN2A1*, *MTA3*, *sax-1* and *xpo1*. The highest number of genes under selection was present in the Norwegian wild tundra reindeer; on the other hand, the Nenets domestic reindeer had the lowest number of genes under selection.

### Divergence time estimations

We inferred the divergence time using the tool Jocx for the following population pairs: the Norwegian wild tundra reindeer vs. the Finnish wild forest reindeer, the Finnish wild forest reindeer vs. the Eveny domestic reindeer, the Norwegian wild tundra reindeer vs. the Eveny domestic reindeer and the Norwegian wild tundra reindeer vs. the Norwegian domestic reindeer using the top 39 scaffolds. The Finnish wild forest reindeer was estimated to have diverged from the Norwegian wild tundra and the Eveny domestic reindeer ~ 51.6 thousand years ago (Kya) and ~ 119.3 Kya, respectively. Similarly, the Norwegian wild tundra reindeer was estimated to have diverged from the Norwegian domestic reindeer and the Eveny domestic reindeer ~ 12.0 Kya and ~ 22.0 Kya, respectively. We found that the divergence between the Finnish wild forest reindeer and the Eveny domestic reindeer (~ 119.3 Kya) occurred earlier than between the Norwegian wild tundra and the Eveny domestic reindeer (22.0 Kya).

## Discussion

We have generated and described here a highly contiguous new genome assembly for the Fennoscandian domestic reindeer (*R. tarandus tarandus*) and used this updated assembly as a reference genome in the population genomic analyses. To date, five reference assemblies for reindeer/caribou have been published (Table 1). With N50 contig and N50 scaffold values of 146.78 kb and 69.739 Mb, respectively, our reindeer assembly represents the most complete reference genome of reindeer. Pairwise genome comparison between our assembly with the cattle reference genome indicated high synteny with 37 reindeer scaffolds mapping to all cattle chromosomes. All the 37 scaffolds are above 10 Mb in size and represent more than 95% of the assembly and, thus, our reference genome is assembled at near-chromosomal level. Here, we have used the updated assembly to perform the most comprehensive population genomic study of the Eurasian reindeer species so far.

Our phylogenetic analyses (Fig. 3) show that the Finnish forest reindeer (*R. t. fennicus*) is genetically distinct from the wild tundra and domestic tundra reindeer in North Europe. Based on the genetic distinctiveness and morphological and ecological differences found between the forest and tundra reindeer^1^, we agree with the conclusion by Harding^29^ that the taxonomical status of ‘subspecies’ is pertinent for the Finnish Forest reindeer and tundra reindeer rather than the taxonomical status of two different ‘eco-types’. Moreover, we present here for the first time an estimate of the subspecies divergence time and found that the northern European *R. t. fennicus* and *R. t. tarandus* diverged ~ 52 Kya. This result indicates that the ‘subspeciation’ had already begun before the ancestral populations of the present-day forest and tundra reindeer colonised Northern Europe after the Last Glacial Maximum, encompassing the period ~ 10–12 Kya^30^. Furthermore, the origins of the ancestral populations that spread to Northern Europe may have been in different refugia populations. Our studies on the genetic structure of the northern Eurasian *Rangifer tarandus* populations point towards three main genetic clusters, suggesting the existence of three ancestral glacial refugia populations (Figs. 2 and 4): The Russian/North American cluster reflecting the Beringian-Eurasian lineage originating from the regions of eastern Siberia‒the ancient Bering Land bridge‒Alaska^14^, the Fennoscandian cluster obviously descending at least partly from the South/Central European refugia populations^7^ and the cluster of the Finnish forest reindeer. The Svalbard reindeer may have descended from the large Beringian-Eurasian glacial population^14^. This conclusion of three main glacial refugia populations is in agreement with the partial mtDNA D-loop sequence analysis of 14 Eurasian and North American domestic and wild *Rangifer tarandus* populations^14^. Interestingly, our phylogenetic and structure analyses showed genetic affinity between the wild Finnish forest reindeer and the domestic forest reindeer from the Zabaikal district, east of Lake Baikal in southern Siberia. This novel finding suggests common ancestries of these two forest reindeer populations in a glacial refugium population. The wild forest reindeer became extinct in Finland in the beginning of the 1900s, and the current population is based on animals that migrated back to eastern Finland from Russian Karelia starting in the 1950s^31^. Archaeo-osteological evidence has indicated that the forest reindeer also originally spread to Finland from the east ~ 7500 years ago, following the retracting ice margin, while the tundra reindeer may have colonised northern Fennoscandia, especially along the Norwegian narrow ice-free coastal zone^32^.

We found with our extended genomic dataset that in both main geographic, genetically distinct population clusters (PCA, phylogenetic tree), there are domestic animals, suggesting at least two independent reindeer domestication processes for the Fennoscandian and Russian domestic reindeer in different parts of Eurasia. This conclusion is also supported by previous microsatellite and mtDNA studies^33^. Our estimate suggests that the Fennoscandian and Russian (the Eveny domestic reindeer analysed here) phylogenetic clusters diverged ~ 22 Kya, while the Russian cluster and the wild Finnish forest reindeer cluster may have separated ~ 119 Kya, providing additional evidence for the genetic distinctiveness of the Finnish forest reindeer in terms of its origin. Moreover, the divergence time estimations suggest that Yakutian Eveny reindeer are taxonomically more like the tundra reindeer subspecies type than they are like forest reindeer. This agrees also with the contemporary herding cultures, where Eveny reindeer nomads migrate still with their reindeer all the way up to the Arctic Ocean (east of the Lena River Delta), in a treeless tundra habitat. Whereas the Evenki with their reindeer practice their semi-nomadic lifestyle still in the more southerly forest areas in Siberia, in South Yakutia, around Lake Baikal (Zabaikal), and all the way into Inner Mongolia. Our estimate indicates that the divergence between the Norwegian wild tundra reindeer and the Norwegian domestic reindeer occurred ~ 12 Kya. However, ancestral wild reindeer populations of Fennoscandian domestic reindeer are not well known, and at least in northernmost Fennoscandia, local wild tundra reindeer may not have been domesticated when the indigenous Sámi people shifted from a livelihood based on reindeer hunting to reindeer pastoralism, starting from the mid-sixteenth century^33,34^. These assumptions about the origins of northern Fennoscandian domestic reindeer are based on archaeological genetic studies on temporal changes and the current distribution of common mtDNA haplogroups in domestic and wild reindeer in Fennoscandia and northwestern Russia (for review see Ref.^33^). However, our whole genome sequencing data of individual animals of three Russian geographic populations and those of the Nenets and Eveny reindeer do not reveal a candidate population whose ancestral population could have been the ancestral population for the Fennoscandian reindeer as well. Despite that, interestingly, our genomic data indicated genetic affinity between the Nenets and Eveny reindeer from northwestern (Arkhangelsk region) and northeastern (Sakha Republic) Russia, respectively. This indicates that reindeer herding and pastoralism have been based on animals of the same genetic origin in a very large geographical area in the northern Russian territory among different northern indigenous societies. As pointed out by Harding et al.^29^, these reindeer breeds belong to the ‘Siberian reindeer’ group of reindeer (*R. t. sibiricus*). This is in line with older anthropological theories of domestication, which assume a domestication cradle of reindeer in South Siberia, from which then the Proto-Samoyeds migrated north-westwards to West Siberia, whereas the ancestors of the Evenki (Tungus) migrated northeast^35^. In contrast to our findings, genotyping of a limited number of autosomal microsatellites in the Russian reindeer breeds^36,37^ clustered the Nenets and Eveny reindeer into different genetic subgroups. The Nenets samples genotyped in the study by Svishchera et al.^37^ were collected in different regions (the Yamal-Nenets and Khanty-Mansi regions) than the Nenets individuals sequenced here. Similarly, Kharzinova et al.^38^ found that the Nenets reindeer from the Arkhangelsk region was not closely related to the Eveny reindeer in Sakha^38^. However, in that study, the authors used Illumina Bovine HD Beadchip to characterise the various reindeer populations, the approach of which is more prone to ascertainment bias.

In the phylogenetic tree based on genetic distances between the individuals (Fig. 3), the Finnish wild forest reindeer individuals are grouped into two closely related branches supported by 100% bootstrap value. This result shows that our samples of the Finnish Forest reindeer were from two geographical conservation areas, Kainuu in eastern Finland and Suomenselkä in Inner Finland. Moreover, our genomic data indicate that the individuals were not genetically influenced by the Finnish domestic reindeer, suggesting that conservation of the Finnish wild forest reindeer has been successful as hybridisation with domestic reindeer may have not happened or is rare.

Although the sample size, sequencing depth and quality of a reference genome may affect the level of genetic diversity seen in animal populations^39^, the within-population genetic diversity estimates presented here (Table 2) indicate that domestic reindeer typically exhibit higher genetic diversity than, for example, domestic cattle breeds (*Bos taurus*)^40^, domestic horse breeds (*Equus ferus caballus*)^41^ and domestic sheep breeds (*Ovis aries*)^38^. Compared to domestic cattle, domestic horse and several other domesticated farm animal species, the domestic reindeer is in the early stage of human-driven domestication. In addition to having a less intensive human-made artificial selection, domestic reindeer populations may have had larger founder population sizes, and possible admixture with wild reindeer populations could have contributed to the level of within-population genetic diversity^7^.

The genome-wide scans for selective sweeps conducted in this study focused on five distinct reindeer subpopulations: Finnish wild forest reindeer, Norwegian wild tundra reindeer, Fennoscandian domestic reindeer (the Finnish and Norwegian populations combined), Nenets domestic reindeer and Eveny domestic reindeer. Mapping the identified selective sweep genomic regions to specific genes showed distinct patterns in each population, with counts of genes under selection being 247, 290, 258, 176 and 271 for the Finnish wild forest reindeer, the Norwegian wild tundra reindeer, the Fennoscandian domestic reindeer, the Nenets domestic reindeer and the Eveny domestic reindeer, respectively (Supplementary Data 4). The varying numbers of genes under selection in the different population groups highlight potential differences in adaptive pressures and genomic responses among these subpopulations. The genes identified under positive selection provide valuable insights into the adaptive processes shaping the genetic makeup of these reindeer populations. Notably, genes related to cold adaptation, non-shivering thermogenesis, smooth muscle contraction, blood pressure regulation, response to temperature, basal metabolic rate and energy metabolism exhibited signs of positive selection in multiple reindeer populations^21^. Additionally, immune response-related genes were highlighted, suggesting the importance of these genes in the context of local environmental challenges^42^. Furthermore, genes associated with ATP, lipid and energy metabolism were under selection, indicating the relevance of these metabolic pathways for reindeer survival and adaptation^7,41,43^. Interestingly, genes linked to calcium binding, calcium metabolism, calcium homeostasis and circadian rhythm also showed signals of positive selection. These findings align with previous studies linking these genes to reindeer-specific characteristics^5^. These genes may play a pivotal role in maintaining physiological functions crucial for survival in the northern environments inhabited by reindeer. An investigation into common genes under selection across all reindeer populations identified 18 genes shared among all five subpopulations. These genes included *Gag*, *Pol*, *RAB9A*, *MAN2A1*, *MTA3*, *sax-1* and *xpo1*. The presence of these shared genes suggests their significance in core adaptive processes across diverse reindeer populations. Interestingly, *gag* and *pol* are two of the three major proteins encoded within the retroviral genome^44^. These genes are considered to play an important role in creating new gene families, the process of which is defined as ‘molecular domestication’. Such phenomena can help the organism adapt to new circumstances. The role of these genes and retrovirus in the adaptation and domestication of reindeer in northernmost Eurasia needs further research (see Chessa et al.^45^). Our results indicate that the selection pressure for these genes was highest in the wild tundra and forest reindeer and in the Nenets and Eveny reindeer, which are less managed by humans, compared to the Fennoscandian domestic reindeer (see also Weldenegodguad et al.^7^, Supplementary Dataset 6).

In the present study we have generated a highly contiguous genome assembly for the Fennoscandian domestic reindeer (*R. t. tarandus*) and utilised it as a reference genome for extensive population genomic analyses. The new assembly demonstrates high quality with contig and scaffold metrics, indicating significant improvements compared to previous reindeer reference genomes. Phylogenetic analyses reveal genetic distinctiveness between Finnish wild forest reindeer and Norwegian wild tundra and domestic reindeer in Northern Europe. The genetic differentiation supports the idea that ‘subspecies’ is a more appropriate taxonomical classification for Finnish wild forest reindeer and Fennoscandian tundra reindeer than is considering them different ‘eco-types’. Notably, genetic affinity was detected between Finnish wild forest reindeer and domestic forest reindeer from southern Siberia, implying shared ancestral populations. Moreover, genetic clusters of domestic animals in both the Fennoscandian and Russian populations suggest at least two independent reindeer domestication processes for these regions. We identified that genes related to retroviral elements (*gag* and *pol*) are common genes under selection across all five groups of the reindeer population. These genes play a role in ‘molecular domestication’, potentially aiding adaptation to new circumstances. These genes show stronger selection pressure in less managed populations and in wild tundra/forest reindeer. Overall, the study provides insights into the complex evolutionary history, domestication, and genetic adaptation of reindeer populations across different regions. It sheds light on the genetic basis of adaptations related to climate, environment and human interaction, opening avenues for further research into the unique features of reindeer in the northernmost parts of Eurasia.

## Materials and methods

### Sample information

Animal handling procedures and sample collections were performed in accordance with the legislation approved by the Animal Experiment Board in Finland (ESAVI/7034/04.10.05.2015) and the Russian Authorisation Board (FS/U.VN-03/163,733/07.04.2016). We confirm that all experiments were performed in accordance with relevant guidelines and regulations, and the study is reported in accordance with ARRIVE guidelines (https://arriveguidelines.org).

In our previous study^7^, we used the Illumina technology and assembled the de novo genome of a one-year-old male reindeer (*R. tarandus tarandus*) from Sodankylä, Finland. In the present study, we improved the genome assembly of this same individual. The DNA for library preparations and sequencing was extracted using a standard phenol–chloroform extraction from liver and muscle samples, which were collected at slaughter and stored in RNA*later* solution (Ambion/QIAGEN, Valencia, CA, USA).

For the resequencing and population genome analyses, we collected samples from Fennoscandian reindeer of the Muddusjärvi and Sallivaara herding cooperatives in northern Finland (n = 10 males, blood samples in EDTA tubes), Nenets reindeer from the Arkhangelsk region in northwest Russia (n = 2 females and 8 males, hair samples), Eveny reindeer from the Eveno-Bytantay region in northern Sakha, Russia (n = 3 females and 6 males, blood samples in EDTA tubes), and wild forest reindeer (*R. t. fennicus*) in Finland (n = 5 females and 1 male, blood samples in EDTA tubes). DNA was extracted using DNeasy Blood and Tissue Kits (Qiagen, Valencia, CA, USA). In addition, we included recently published data on 23 *Rangifer* genomes^7^ in this study: domestic forest reindeer from Russia (n = 2), domestic tundra reindeer from Norway (n = 4), wild tundra reindeer from Russia (n = 2), wild tundra reindeer from Russia (n = 4), wild tundra reindeer from Norway (n = 5), wild arctic reindeer from Svalbard, Norway (n = 3), Alaskan domestic reindeer from the USA (n = 2) and Alaskan wild caribou from the USA (n = 1). More detailed information of these animals and DNA extraction of the samples are given in Flagstad and Røed.^14^.

### Library preparation and sequencing

Four Chicago and HiC libraries were prepared^9^ in the Dovetail laboratory. For each library, ~ 500 ng of gDNA (mean fragment length = 70 kb) was reconstituted into chromatin in vitro and fixed with formaldehyde. Fixed chromatin was digested with DpnII, the 5’ overhangs were filled in with biotinylated nucleotides and then the free blunt ends were ligated. After ligation, crosslinks were reversed, and the DNA was purified from protein. The purified DNA was treated to remove biotin that was not internal to the ligated fragments. The DNA was then sheared to ~ 350 bp mean fragment size, and sequencing libraries were generated using NEBNext Ultra enzymes and Illumina-compatible adapters. Biotin-containing fragments were isolated using streptavidin beads before PCR enrichment of each library. The libraries were sequenced on an Illumina HiSeq X platform.

Two Dovetail HiC libraries were prepared in a comparable manner, as described previously^46^. For each library, chromatin was fixed in place in the nucleus with formaldehyde and then extracted. Fixed chromatin was digested with DpnII, the 5’ overhangs were filled in with biotinylated nucleotides and then the free blunt ends were ligated. After ligation, crosslinks were reversed, and the DNA was purified from protein. Purified DNA was treated to remove biotin that was not internal to the ligated fragments. The DNA was then sheared to ~ 350 bp mean fragment size, and sequencing libraries were generated using NEBNext Ultra enzymes and Illumina-compatible adapters. Biotin-containing fragments were isolated using streptavidin beads before PCR enrichment of each library. The libraries were sequenced on an Illumina HiSeq X platform.

The de novo assembly shotgun reads, Chicago library reads and Dovetail HiC library reads were used as input data for HiRise, a software pipeline designed specifically for using proximity ligation data to scaffold genome assemblies^9^. An iterative analysis was conducted. First, shotgun and Chicago library sequences were aligned to the draft input assembly using a modified SNAP read mapper^47^. The separations of Chicago read pairs mapped within draft scaffolds were analysed by HiRise to produce a likelihood model for genomic distance between read pairs, and the model was used to identify and break putative misjoins, score prospective joins and make joins above a threshold. After aligning and scaffolding the Chicago data, Dovetail HiC library sequences were aligned and scaffolded following the same method. After scaffolding, shotgun sequences were used to close gaps between contigs.

### Topologically associated domains (TAD) analysis

Hi-C contact matrices in two formats, namely cool and hic, were generated. Both contact matrices were generated from the same BAM file by using read pairs where both ends were aligned with a mapping quality of 60. TADs were identified using the Arrowhead program implemented in the Juicertools package^48^. We called TADs at three different resolutions: 10 kbp, 25 kbp and 50 kbp. The parameters used were -k KR -m 2000 -r 10,000, -k KR -m 2000 -r 25,000 and -k KR -m 2000 -r 50,000. A/B compartments were identified at 1 Mbp using the eigenvector program implemented in the Juicertools package. The parameters used were KR BP 1,000,000. Isochores were predicted using the isofinder program^49^. The parameters used were 0.90 p2 3000. The output was postprocessed to convert it to a bedpe format. CTCF sites were predicted using the CREAD program^50^. The position weight matrix was downloaded from the CTCFBSDB 2.0 website. The output was then postprocessed to convert it to a BED file. Multires files were generated using the clodius package. These files can be loaded in HiGlass, an open-source visualisation tool^51^.

### Genome annotation

Repeat families found in the genome assemblies of *Rangifer tarandus* were identified de novo and classified using the software package RepeatModeler (Version 2.0.1) (http://www.repeatmasker.org/RepeatModeler/). RepeatModeler depends on the programs RECON (Version 1.08) and RepeatScout (Version 1.0.6) for the de novo identification of repeats within the genome. The custom repeat library obtained from RepeatModeler were used to discover, identify and mask the repeats in the assembly file using RepeatMasker (Version 4.1.0)^52^.

Coding sequences from *Bos taurus*, *Rangifer tarandus* (http://www.caribougenome.ca/downloads)^6^, *Rangifer tarandus* (in Chinese, http://animal.nwsuaf.edu.cn)^4^, Ovis* aries* and *Capra hircus* were used to train the initial ab initio model for *Rangifer tarandus* using the AUGUSTUS software (Version 2.5.5)^53^. Six rounds of prediction optimisation were done with the software package provided by AUGUSTUS. The same coding sequences were also used to train a separate ab initio model for *Rangifer tarandus* using SNAP (Version 2006–07-28)^54^. RNA-Seq data from four adipose tissues were used for improving the annotation. RNA-seq reads were mapped onto the genome using the STAR aligner software (Version 2.7)^55^ and intron hints generated with the bam2hints tools within the AUGUSTUS software. MAKER^56,57^, SNAP and AUGUSTUS (with intron–exon boundary hints provided from RNA-seq) were then used to predict genes in the repeat-masked reference genome. To help guide the prediction process, Swiss-Prot peptide sequences from the UniProt database were downloaded and used in conjunction with the protein sequences from *Bos taurus*, *Rangifer tarandus* (http://www.caribougenome.ca/downloads), *Rangifer tarandus* (in Chinese, http://animal.nwsuaf.edu.cn/), *Ovis aries* and *Capra hircus* to generate peptide evidence in the Maker pipeline. Only genes that were predicted by both the SNAP and AUGUSTUS software were retained in the final gene sets. To help assess the quality of the gene prediction, AED scores were generated for each of the predicted genes as part of the MAKER pipeline. Genes were further characterised for their putative function by performing a BLAST search of the peptide sequences against the UniProt database. tRNA sequences were predicted using the software tRNAscan-SE (Version 2.05)^58^.

### Population genomic data analysis

For the various population genomic analyses, we had whole genome sequencing data of 58 *Rangifer* individuals (Supplementary Data 2): 23 genomes from our previous study^7^ and a new set of 35 individuals sequenced in the present study. Whole genome sequencing of DNA samples of these new individuals was done in BGI, as previously described^7^. The whole dataset (58 genomes) was used only in the PCA and in the individual-based phylogenetic analysis, while more comprehensive population genomic analyses were done for seven populations in which genomes of several individuals were sequenced. These populations were the Finnish domestic reindeer (Fi-D-R, n = 10), the Norwegian domestic reindeer (No-D-R, n = 4), the Norwegian wild tundra reindeer (No-W-T, n = 5), the Finnish wild forest reindeer (Fi-F-R, n = 6), the Nenets domestic reindeer from the Arkhangelsk region (Ar-D-R, n = 10), the Eveny domestic reindeer from Sakha (Yakutia) (Ya-D-R, n = 9) and the wild arctic Svalbard reindeer (Sv-W-A, n = 3). Our new and improved reindeer genome assembly was used as a reference in the population genomic analyses.

### Quality control and preprocessing

The quality of the raw read data was inspected using FastQC software (Version 0.11.8)^59^. MultiQC (Version 1.8.dev0)^60^ was used for generating quality control reports of all samples.

### Alignment

Samples were aligned with BWA (Version 0.7.17-r1188)^61^ against the assembled reindeer reference genome using default parameters. Before the alignment, the contig names in the reference were modified to be in line with SAM format. Resulting BAM files were sorted and indexed using SAMTools (Version 1.9)^62^. Duplicate alignments were marked with PicardTools (Version 2.18.16, https://broadinstitute.github.io/picard/).

### Variant calling

SNPs and indels were called according to the GATK best practice guidelines using GATK (Version 4.0.11)^63^. First, HaplotypeCaller was used for calling variant from individual duplicate-marked alignment files. Per-sample gVCF files produced by HaplotypeCaller were combined into a multisample gVCF file using the CombineGVCFs tool. The GenotypeGVCFs tool was then used for joint genotyping of all samples. Separate SNP and indel VCF files were generated for the plotting of quality scores to select appropriated thresholds for hard filtering of the variants. Based on the plots and the recommendations in the GATK 4 user guide, the following filters were applied to the variant set: FS > 60.0, MQ < 40.0, MQRankSum < -8.0, QD < 2.0, ReadPosRankSum < -8.0 and SOR > 3.0 for SNPs and FS > 200.0, MQ < 40.0, QD < 2.0, ReadPosRankSum < -8.0 and SOR > 5.0 for indels. Variants that passed all filters were extracted using the SelectVariants tool to create the final high-quality set of variants. Variant call statistics were generated using bcftools (Version 1.9)^64^ and MultiQC (Version 1.8.dev0)^60^.

### Principal component analysis (PCA)

PCA was performed with the R^65^ package SNPRelate (Version 1.18.1)^66^ using the filtered variant data as input. LD-based pruning using the snpgdsLDpruning function was first applied to avoid strong influence of linked SNP clusters. Two different LD thresholds, 0.2 and 0.5, were used for filtering the SNP set. The snpgdsPCA function was then utilised for plotting the PCA results.

In an alternative approach, genotype probabilities rather than called genotypes were calculated using ANGSD software (Version 0.929)^67^ using the following parameters: -uniqueOnly 1 -remove_bads 1 -only_proper_pairs 1 -trim 0 -C 50 -baq 1 -minMapQ 20 -minQ 20 -minInd 29 -setMinDepth 5 -setMaxDepth 100 -doCounts 1 -GL 1 -doMajorMinor 1 -doMaf 1 -skipTriallelic 1 -SNP_pval 1e-3 -doGeno 8 -doPost 1. Sites totalling 254,891 were retrieved and used for calculating pairwise genetic distance using ngsDist from ngsTools (Version 1.0.2)^68^.

### Population diversity statistics

Population diversity statistics were calculated from the SNP data using the R package PopGenome (Version 2.7.1)^69^. The analysed SNP data consisted of the SNPhylo-filtered set of SNPs as described in the section of this study titled ‘Phylogenetic analysis’. For the seven main populations (Fi-D-R, No-D-R, No-W-T, Fi-F-R, Ar-D-R, Ya-D-R and Sv-W-A), the average pairwise nucleotide diversity within a population (π) and the proportion of polymorphic sites (Watterson’s θ) were calculated from the SNP data using the Bio::PopGen::Statistics package in BioPerl (Version 1.6.924)^70^. Moreover, average minor allele frequencies of SNPs were calculated for those seven main populations in addition to counts of population-specific, private SNPs. F_ST_ and πi values were also calculated for consecutive windows of 50 K SNPs, and average values of SNPs within the windows were plotted. A similar analysis of site frequency spectrum values was conducted.

### Phylogenetic analysis

An NJ tree of all samples was generated from the SNP data using SNPhylo (Version 20,160,204)^71^. First, the SNPs were further filtered with SNPhylo using the following filter thresholds: minimum depth of coverage > 5, the percentage of low-coverage samples < 5%, the percentage of samples with no SNP information < 5%, LD < 0.1 and minor allele frequency > 0.05. SNPhylo-generated sequences from the SNP data were used to perform multiple alignment of the sequences. PHYLIP tools^72^ were then used for computing a protein distance matrix and creating an NJ tree. FigTree (Version 1.4.4)^73^ was used for enhancing the tree appearance. An NJ tree with bootstrap values was generated with PHYML 3.0 software, using the multiple sequence alignments from SNPhylo as input. One hundred bootstrap replicates were generated, and the obtained bootstrap tree was exported in Newick format to FigTree, which was again used for enhancing the tree appearance and adding the bootstrap support values to the branches.

For the population-level NJ tree, average pairwise F_ST_ values of the seven main populations (Fi-D-R, No-D-R, No-W-T, Fi-F-R, Ar-D-R, Ya-D-R and Sv-W-A) were computed using vcftools (Version 0.1.13)^74^. A distance matrix of the pairwise values was generated, and the matrix was used for building an NJ tree using MEGA7 software^75^.

### Population structure analysis

Population admixture analysis was performed using ADMIXTURE software (Version 1.3)^76^. Hard-filtered SNP data was first converted into the binary PLINK format using PLINK (Version 1.07)^77^, after which 500,000 randomly sampled SNPs were extracted for the analysis. ADMIXTURE was then run using different K values ranging from 2 to 12 and with the bootstrap parameter set to 200 replicates for estimation of standard errors. Population structure plots were generated using the R package pophelper^78^.

### Positive selective sweep analysis

Genomic scans for positive selective sweeps were performed with RAiSD (Version 2.9)^20^ using default parameters. RAiSD calculates the μ-statistic, a composite evaluation test that scores genomic locations by quantifying changes in the site frequency spectrum (SFS), the levels of LD and the amount of genetic variation along the chromosome^20^. In the analysis, we pooled the datasets of the two Fennoscandian domestic reindeer populations (the Finnish and Norwegian reindeer) to improve the statistical power of the selective sweep analysis. Our PCA and phylogenetic analyses (see Figs. 2 and 3) showed close genetic affinity between these reindeer populations inhabiting similar biogeographic northern regions. We performed a selective sweep analysis for each of the five subpopulations (wild Finnish forest reindeer, wild Norwegian tundra reindeer, Fennoscandian domestic reindeer, Nenets domestic reindeer and Eveny domestic reindeer) (Supplementary Data 4). We selected scaffold length > 9 Mb (top 40 scaffold) for selective sweep analysis, and RAiSD was run separately for each scaffold. To detect highly supported sweeps, we focused on each scaffold with the top 1% of the μ-statistic. The cutoff value for μ-statistics was taken as the 99.99th percentile of the empirical distribution across the genome for each scaffold. Finally, the outlier selective sweep regions were manually annotated with the reference annotation (.gtf) file using BedTools (Version 2.29.0)^79^.

### Estimation of split time between different reindeer ecotypes under the coalescent hidden Markov model (CoalHMM) Framework

The divergence times between different reindeer ecotypes were estimated using the CoalHMM implemented in Jocx^80^. To estimate the split time, we selected tundra and forest reindeer ecotypes from four main clusters based on the PCA plot (Fi-F-R, No-W-T, No-D-R and Ya-D-R; see Fig. 2) and estimated divergence times between the population pairs: No-W-T vs. Fi-F-R, Fi-F-R vs. Ya-D-R, No-W-T vs. Ya-D-R and No-W-T vs. No-D-R. For the analysis, we selected a sample with the highest coverage from each population (NMBU-33, NMBU-39, RTF96 and YR6) and, using the BAM mapping files, generated a consensus pseudogenome for each sample using ANGSD (Version 0.931)^67^ (*-doFasta 3*). In ANGSD, we used *-minMapQ 30* to discard reads with a minimum mapping quality lower than 30. Additional filter parameter (*-uniqueOnly 1*) was also used to discard reads that did not map uniquely. We split the pseudogenomes into nonoverlapping, 10 Mbp segments using seqkit^81^. In each genome, we identified a total of 232 nonoverlapping, 10 Mbp segments across the top 39 scaffolds (scaffold length greater than 10 Mb). A maximum likelihood estimation of the divergence time was then performed independently based on each 10 Mbp segment using an isolation model from the tool Jocx (https://github.com/jade-cheng/Jocx). The demographic model we used in this analysis is an isolation model without gene flow after split. We used a mammalian mutation rate of 2.2 × 10^−9^ per base per year^82^ to rescale the estimated model parameter to years.

### Supplementary Information

Supplementary Information 1.

Supplementary Information 2.

Supplementary Information 3.

Supplementary Information 4.

Supplementary Information 5.

Supplementary Information 6.

## Data Availability

Our assembly is available under ENA assembly accession: PRJEB65321 and WGS data of 35 new samples are available under ENA study accession: PRJEB65932.
